# GTB – an online genome tolerance browser

**DOI:** 10.1186/s12859-016-1436-4

**Published:** 2017-01-06

**Authors:** Hashem A. Shihab, Mark F. Rogers, Michael Ferlaino, Colin Campbell, Tom R. Gaunt

**Affiliations:** 1 MRC Integrative Epidemiology Unit (IEU), University of Bristol, Bristol, BS8 2BN UK; 2 Intelligent Systems Laboratory, University of Bristol, Bristol, BS8 1UB UK

**Keywords:** SNVs, Mutation, Pathogenicity prediction, Prediction algorithm, Variant effect prediction, Genome browser, Genome tolerance

## Abstract

**Background:**

Accurate methods capable of predicting the impact of single nucleotide variants (SNVs) are assuming ever increasing importance. There exists a plethora of *in silico* algorithms designed to help identify and prioritize SNVs across the human genome for further investigation. However, no tool exists to visualize the predicted tolerance of the genome to mutation, or the similarities between these methods.

**Results:**

We present the Genome Tolerance Browser (GTB, http://gtb.biocompute.org.uk): an online genome browser for visualizing the predicted tolerance of the genome to mutation. The server summarizes several *in silico* prediction algorithms and conservation scores: including 13 genome-wide prediction algorithms and conservation scores, 12 non-synonymous prediction algorithms and four cancer-specific algorithms.

**Conclusion:**

The GTB enables users to visualize the similarities and differences between several prediction algorithms and to upload their own data as additional tracks; thereby facilitating the rapid identification of potential regions of interest.

**Electronic supplementary material:**

The online version of this article (doi:10.1186/s12859-016-1436-4) contains supplementary material, which is available to authorized users.

## Background

The rate at which single nucleotide variants (SNVs) are being identified across the genome has increased owing to technological advances and the falling costs in whole-genome sequencing [21]. The main challenge facing clinicians and researchers is identifying which of these SNVs contribute to disease predisposition [6]. There are many algorithms capable of predicting the functional consequences of these variants, including those focussing on nonsynonymous SNVs (nsSNVs) that induce amino acid substitutions [4, 18], SNVs that influence specific diseases such as cancer [7, 17], or SNVs that fall within non-coding regions of the genome [8, 14, 19]. However, each method employs a different approach to variant effect prediction, which can sometimes lead to conflicting predictions for the same variant being made. For example, sequence-based algorithms begin with a multiple sequence alignment between the gene or protein of interest and homologous sequences. Here, it is assumed that conserved positions within the alignment indicate that there are strong selective pressures acting on particular residues; therefore, genomic variants occurring at these positions are often considered to be functional. On the other hand, structure-based algorithms use structural properties, such as the accessible solvent area, to identify putative functional variants. These algorithms assume that variants falling at specific sites are functional regardless of sequence conservation, e.g. buried residues. Recently, a new class of prediction algorithms capitalizing on state-of-the-art machine learning paradigms have emerged. These algorithms combine several sequence and structure-based annotations to train classifiers using known disease-causing variants and neutral polymorphisms. A comprehensive review on the underlying methodology of prediction algorithms is given in Ng and Henikoff [12], and a comprehensive comparative evaluation of algorithm performance has been performed by Thusberg et al [22].

The wealth of available prediction algorithms makes assessing the predicted impact of genomic variants a tedious and time consuming task. As a result, databases such as the dbNSFP [9] and the dbWGFP [24] have begun to collate the output of several different prediction algorithms; thereby allowing users to assess the concordance between prediction algorithms. While the reported correlation between existing algorithms varies considerably, ranging from near zero to near perfect correlation [10], no tool exists for visualizing these similarities and differences. In this work, we present the Genome Tolerance Browser (GTB): an online browser for visualizing the predicted tolerance of the genome to mutation and for identifying potential similarities and subtle differences between *in silico* prediction algorithms.

## Construction and content

### Prediction algorithms and conservation scores

We obtained exome-wide pre-computed predictions for 12 non-synonymous computational prediction algorithms, including SIFT [11] and PolyPhen-2 [1], from dbNSFP (version 3.1; [10]). Although dbNSFP includes predictions from a number of genome-wide prediction algorithms and conservation scores, e.g. CADD [8] and GERP++ [3], these predictions are limited to just the coding regions of the human genome. Therefore, we enhanced this dataset to include the non-coding regions of the genome wherever possible. In addition to genome-wide predictions, we obtained exome-wide predictions from two cancer-specific algorithms: FATHMM [17] and TransFIC [7]. The composition of prediction algorithms included in the GTB is summarized in Table 1.

**Table 1:** List of *in silico* prediction algorithms and conservation scores summarized through the Genome Tolerance Browser

**Non-Synonymous Prediction Algorithms**	
**Generic**	**Cancer-Specific**
SIFT	TransFIC (SIFT)
PolyPhen-2 (HumVar & HumDiv)	TransFIC (PolyPhen-2)
MutationAssessor	TransFIC (MutationAssessor)
FATHMM (Unweighted & Weighted)	FATHMM (Cancer)
FATHMM-MKL (Coding)	
MutationTaster2	
PROVEAN	
VEST	
LRT	
MetaLR	
MetaSVM	
**Genome-Wide Prediction Algorithms**	
CADD	
DANN	
FATHMM-MKL (Non-coding)	
fitCons	
**Conservation Scores**	
PhastCons (46-Way)	
PhyloP (46-way; vertebrate, primates and placental mammals)	
PhastCons (100-Way)	
PhyloP (100-way; vertebrate, primates and placental mammals)	
GERP++	
SiPhy	

### Calculating genome tolerance

One of the main objectives of the GTB is to visualize the predicted tolerance of the genome to mutation. To this end, we summarize and normalize predictions from each method at the individual base level as follows: we first permute each base and obtain the corresponding predictions from each algorithm. Where a mutation affects multiple transcripts (within coding regions), we obtain multiple scores per permutation. We normalize these scores so that they fall between 0 and 1 using the following formula:

$$ x=\frac{\left(x- min\right)}{\left( max- min\right)} $$

where *min* and *max* are the lower and upper bounds of the prediction algorithm. Finally, we average these scores across permutations to obtain the overall predicted tolerance of the position to mutation: higher scores indicate that a position is less tolerant to mutation whereas lower scores indicate those that are more tolerant to mutation. We stress that these scores are not new or “transformed” predictions per se, but instead these scores represent the overall tolerance of a particular position to mutation as predicted by the associated in silico algorithms, i.e. on average, how tolerant is the position to mutation. It should be noted that a large proportion of prediction algorithms do not consider variants outside of SNVs, e.g. insertions and deletions, nor do they distinguish between gain-of-function and loss-of-function mutations.

### Visualization

A web-based version of the GTB is available at *http://gtb.biocompute.org.uk* and has been built on top of the Dalliance genome browser [5]. By default, tracks representing two popular non-synonymous prediction algorithms: SIFT and PolyPhen-2, and two genome-wide prediction algorithms: FATHMM-MKL and CADD, are displayed. Using the available options, users can add additional tracks representing a plethora of computational prediction algorithms (see Table 1 for a full list of available methods), or even upload custom annotation data in either *bigWig* or *bigBed* format. The appearance of these tracks can be customized, and publication quality images can be exported in either *SVG* or *PNG* format. Users can also download the entire GTB database or extract GTB scores for specific regions by following the instructions given on the website.

## Utility

In the following section, we demonstrate how the GTB can be used to visualize, compare and contrast several prediction algorithms. Understanding why various algorithms agree in particular regions, but disagree in other regions, is an important aspect when interpreting computational predictions. In addition, when multiple algorithms all yield different predictions and/or tolerance profiles, this could suggest that variants falling in these regions are much harder to predict. Therefore, users should treat predictions with caution and not rely on a single algorithm for interpretation. Further, the browser can also be used to identify potential “regions of interest”. Here, long stretches of intolerance predicted by multiple algorithms may indicate regions worth exploring through in vitro experimentation.

### Visualizing the characteristics of sequence- and structure-based prediction algorithms

**Fig. 1:**
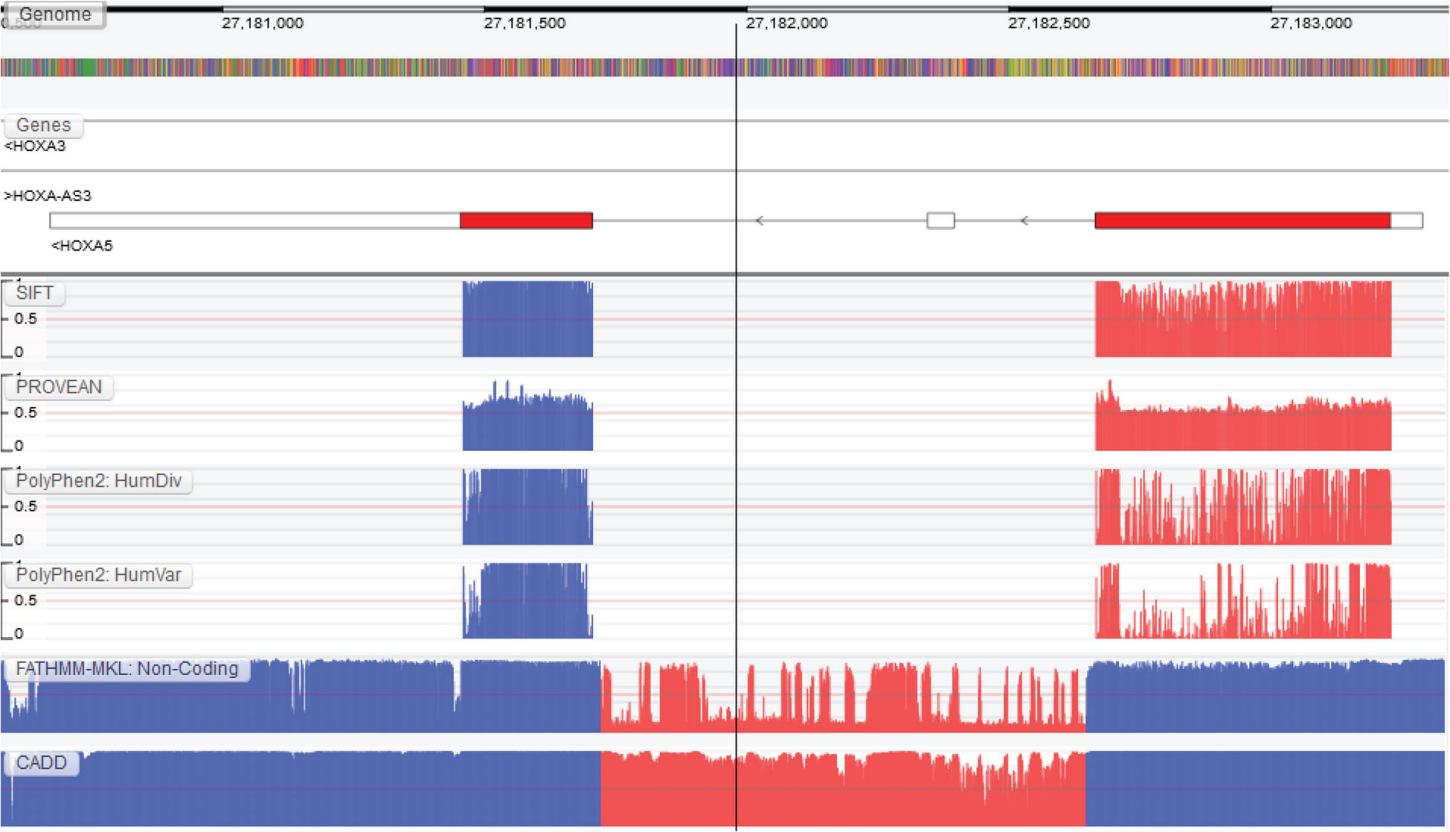
Tolerance profile of *HOXA5* shows regions of similarity between sequence-based prediction algorithms: SIFT and PROVEAN. However, subtle differences in tolerance can be observed when comparing these sequence-based algorithms with a structure-based algorithm, PolyPhen-2. Insight into potential regions of interest can be also obtained from genome-wide prediction algorithms such as FATHMM-MKL and CADD

Figure 1 (see Additional file 1 for larger high resolution images) shows the tolerance profile for *HOXA5*, a member of the *Homeobox* gene cluster, as predicted by two sequence-based algorithms, SIFT [11] and PROVEAN [2], a structure-based algorithm, PolyPhen-2 [1], and two genome-wide prediction algorithms, FATHMM-MKL [19] and CADD [8]. While there are a number of sequence- and structure-based prediction algorithms available in the GTB, these algorithms were chosen due to their use and general popularity in the scientific literature.

Although SIFT shows higher intolerance across *HOXA5*, the overall profile shows similar regions of intolerance to that of PROVEAN. For example, both appear to show high intolerance towards the end of the 1^st^ exon (see region highlighted in red). However, this comes as no surprise given that these genes play a crucial role during embryonic development and are highly conserved across great evolutionary distances [16]. In contrast, PolyPhen-2, which incorporates structure-based properties for variant prioritization, shows a different tolerance profile. Here, it appears that it is specific regions of *HOX5A* that are intolerant to mutation. This suggests that these regions may harbour important structural constraints which are potentially missed when using a pure sequence-based approach. Both PolyPhen-2 models, HumVar and HumDiv, share large regions of similarity (highlighted in red). However, this also comes as no surprise as they both utilize the same underlying prediction algorithm but are trained using slightly different training data [4]. Peaks of predicted intolerance can also be observed across the non-coding region of *HOXA5* when using genome-wide prediction algorithms such as FATHMM-MKL and CADD; thereby suggesting that these regions could also be functional. However, it is interesting to note that FATHMM-MKL appears to give much more granular peaks across the region than CADD. Both algorithms are trained using similar genomic annotations. Therefore, this observation appears to suggest that these algorithms may place greater emphasis on different genomic annotations across *HOXA5*.

**Fig. 2:**
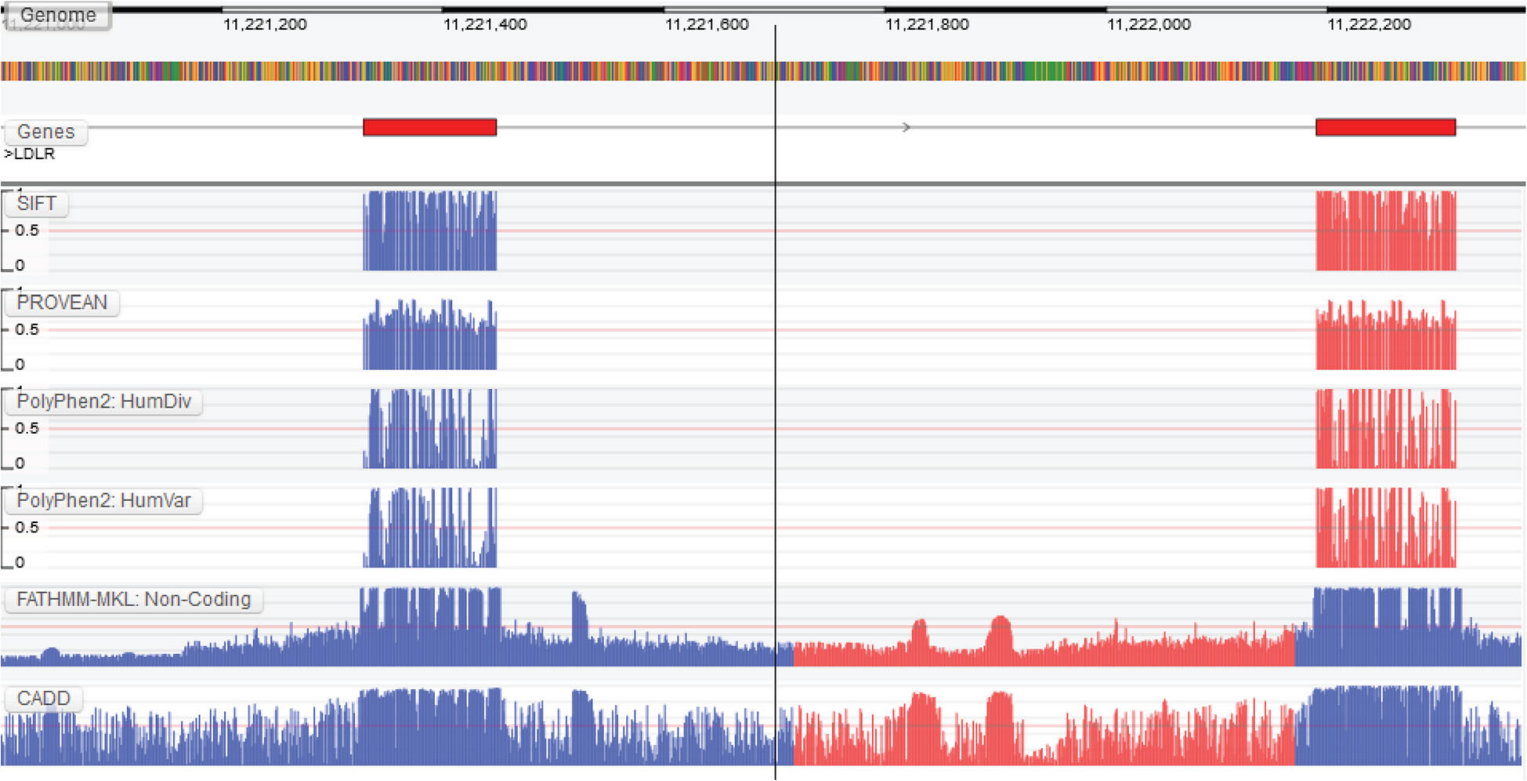
A similar trend in intolerance can be observed across *LDLR* using sequence- and structure-based prediction algorithms, i.e. sequence-based methods tend to agree on intolerance given that they both rely on sequence conservation whereas structure-based algorithms utilize the additional structure-based properties made available to them to show a different tolerance profile. Unlike *HOXA5*, genome-wide prediction algorithms appear to agree on potential peaks of intolerance across the non-coding region of *LDLR*

A similar trend can also be observed across *LDLR*: where variants in this gene have been linked with the autosomal dominant disorder, familial hypercholesterolemia [20]. Here, both SIFT and PROVEAN show similar patterns of intolerance given that they depend solely on sequence conservation for prediction whereas PolyPhen-2 shows a more refined intolerance profile (Fig. 2). These differences could be explained by structural constraints that are potentially missed when using sequence conservation alone, or a larger dependency on structure-based annotations, e.g. the accessible solvent area, across the region. Unlike *HOXA5*, FATHMM-MKL and CADD, are much more similar across the non-coding regions of *LDLR*, which suggests that both algorithms could be relying on the same genomic annotations across this region.

### Visualizing the impact of cancer-specific training

**Fig. 3:**
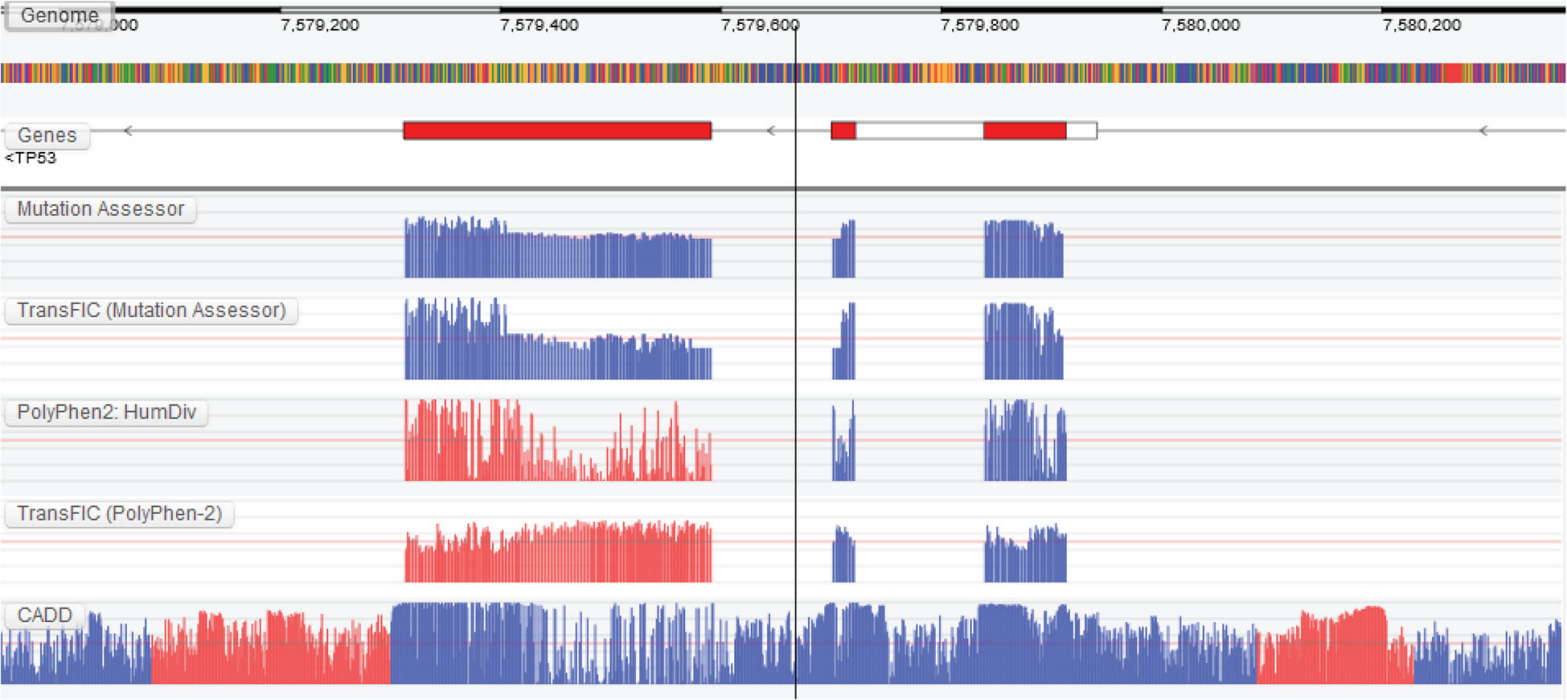
Subtle differences between generic and cancer-specific prediction algorithms can be observed across *TP53*. For example, cancer-specific transformations of traditional germline prediction algorithms amplify intolerance across the entire region

**Fig. 4:**
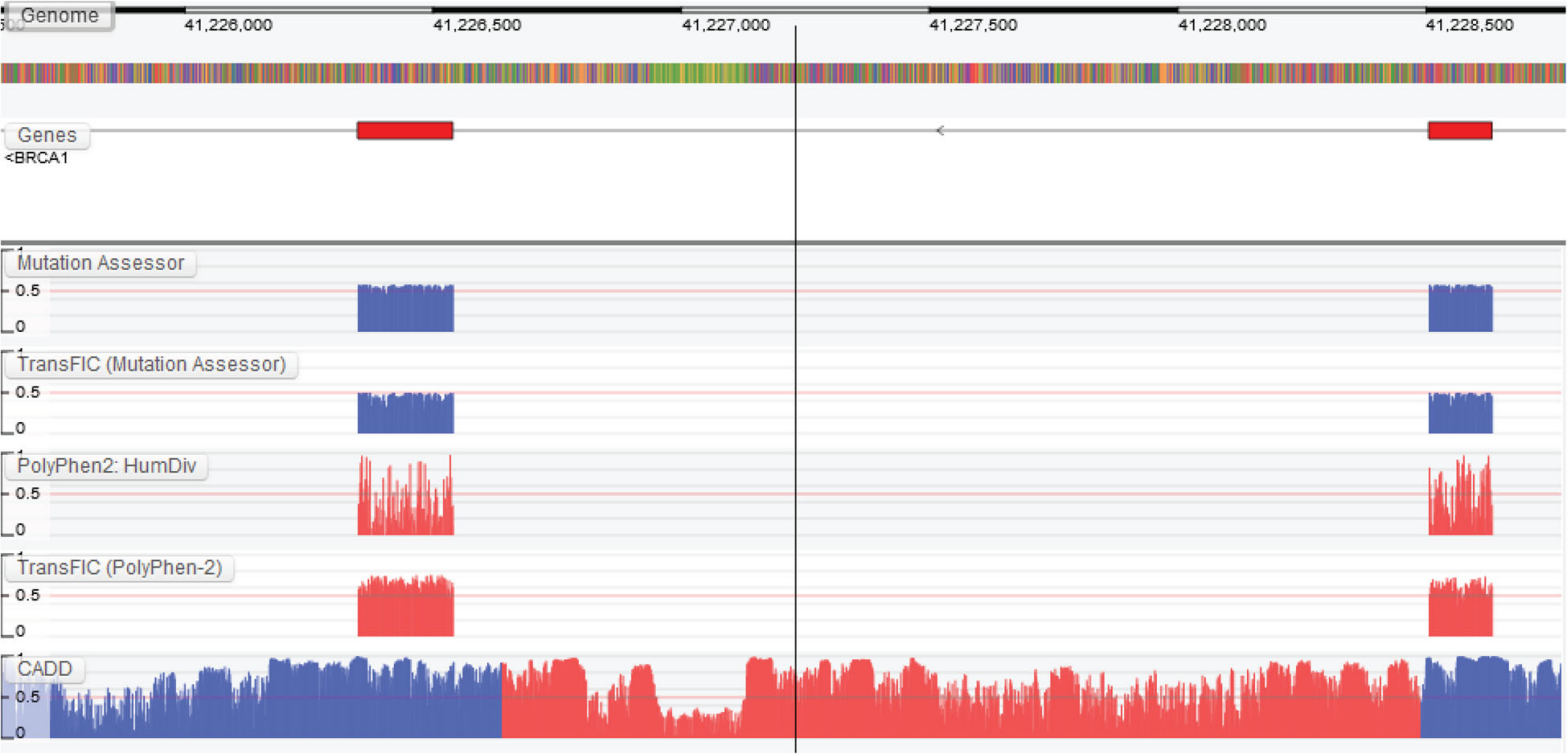
Cancer-specific transformations of traditional germline prediction algorithms amplifies the intolerance of *BRCA1*

Next, we illustrate how the GTB can be used to visualize the differences between traditional and cancer-specific prediction algorithms. Figure 3 shows the tolerance profile for the initial three coding exons of the tumour suppressor gene *TP53* whereas Fig. 4 shows the intolerance profile for the largest exon in *BRCA1. TP53* and *BRCA1* both play a pivotal antiproliferative role and mutations within it predispose individuals to a wide spectrum of early-onset cancers [13, 23]. While traditional germline algorithms such as PolyPhen-2 and MutationAssessor [15] are capable of identifying localized regions of intolerance, cancer-specific transformations of these algorithms [7] are capable of capturing the importance of the entire region with respect to cancer. As a result, the entire region is amplified compared to their original counterparts (e.g. see the PolyPhen-2 region highlighted in red). These amplifications could be the direct result of the cancer-specific training employed in these methods, i.e. these methods are specifically trained to discriminate between cancer-associated variants and all other variants (both germline disease mutations and neutral polymorphisms). Small peaks of predicted intolerance can also be observed in non-coding regions when using genome-wide prediction algorithms (highlighted in red). However, it should be noted that these genome-wide predictions were trained on germline mutations and not cancer-associated mutations. Therefore, the ability of these methods to detect intolerance with respect to cancer remains to be seen. Once again, while there are multiple algorithms in the GTB, we selected the above algorithms due to their overall use and popularity in the scientific literature when predicting the effects of cancer-associated variants.

## Discussion

The Genome Tolerance Browser (GTB) offers a platform to effectively compare and visualize differences in functional predictions between a wide range of algorithms at (or below) the gene level. This enables the researcher to clearly understand the nature of differences in performance and make a more informed decision about the best algorithm to use for a particular scenario. For example, the browser can be used to identify cases in which particular algorithms place greater emphasis on similar annotations during prediction, as illustrated by the emphasis on sequence conservation we observed when comparing SIFT and PROVEAN. The GTB can also be used to detect subtle differences between prediction algorithms. For example, we observed clear discrepancies in predicted intolerance between generic prediction algorithms and cancer-specific prediction algorithms across cancer-associated regions of the genome, illustrating that these different methodologies place greater emphasis on different annotations during prediction.

The potential utility of the GTB goes beyond simply visualizing computational prediction algorithms. For example, other research questions that could be asked include: are prediction algorithms affected by genomic annotations such as open chromatin, transcription factor binding sites and histone modifications; and can some of the observed variability between prediction algorithms be explained by these annotations; given specific genomic annotations, under what circumstances should we use particular prediction algorithms (or particular methodologies towards prediction)?

Finally, the GTB can be used to identify potential regions of interest across the genome, e.g. long stretches of predicted intolerance. In future releases, we plan on developing algorithms for automatically detecting and characterizing these regions of interest.

## Conclusions

The GTB is a visualization platform that enables users to compare a range of existing variant effect prediction algorithms (and other data as additional tracks) in specific regions of the human genome. The GTB enables differences in prediction to be evaluated and facilitates rapid identification of potential regions of interest.

## Availability and requirements

The GTB is freely available online at http://gtb.biocompute.org.uk and the source code for local hosting is available at https://github.com/HAShihab/gtb.

## Additional file

Additional file 1: High resolution versions of Figs. 1, 2, 3 and 4. (DOCX 823 kb)

## Abbreviations

GTB: Genome tolerance browser
nsSNVs: non-synonymous single nucleotide variant
PNG: Portable network graphics
SNV: Single nucleotide variant
SVG: Scalable vector graphics
