# Supplementary material for: GTB – an online genome tolerance browser
Source: BMC Bioinformatics. 2017 Jan 6;18:20. doi: 10.1186/s12859-016-1436-4 (PMC5219737; doi:10.1186/s12859-016-1436-4)
Supplement: Additional file 1: — High resolution versions of Figs. 1, 2, 3 and 4. (DOCX 823 kb) [file 12859_2016_1436_MOESM1_ESM.docx]

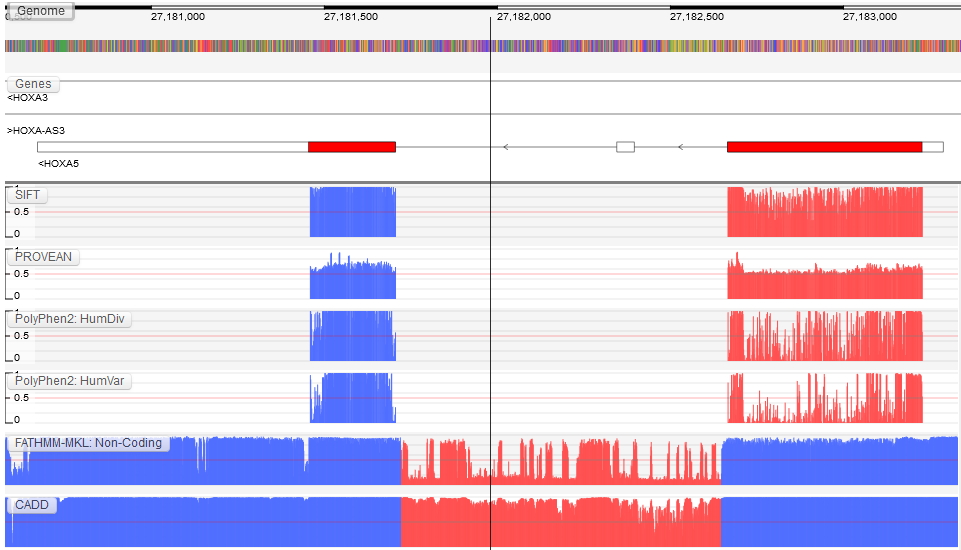


**Figure 1. Tolerance profile of HOXA5 shows regions of similarity between sequence-based prediction algorithms: SIFT and PROVEAN. However, subtle differences in tolerance can be observed when comparing these sequence-based algorithms with a structure-based algorithm, PolyPhen-2. Insight into potential regions of interest can be also obtained from genome-wide prediction algorithms such as FATHMM-MKL and CADD.**


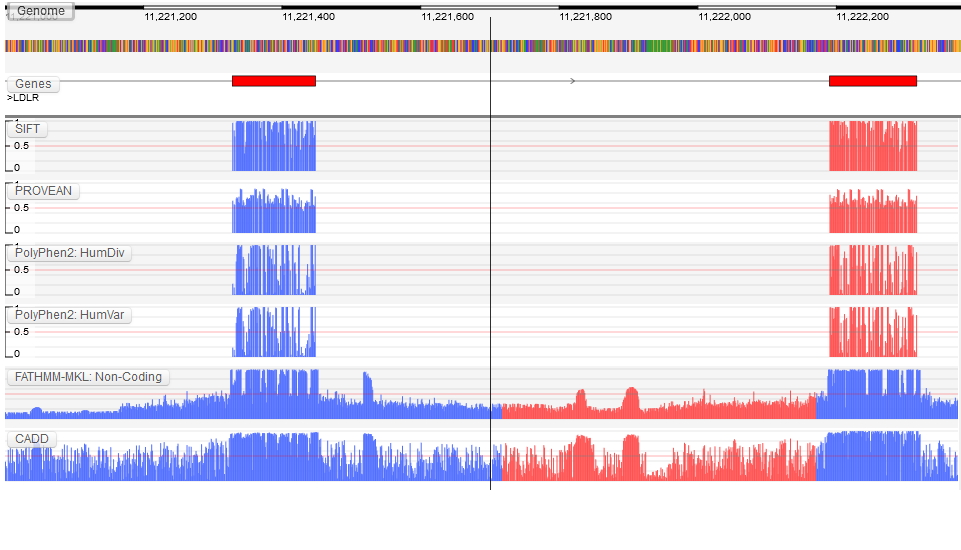


**Figure 2. A similar trend in intolerance can be observed across LDLR using sequence- and structure-based prediction algorithms, i.e. sequence-based methods tend to agree on intolerance given that they both rely on sequence conservation whereas structure-based algorithms utilize the additional structure-based properties made available to them to show a different tolerance profile. Unlike HOXA5, genome-wide prediction algorithms appear to agree on potential peaks of intolerance across the non-coding region of LDLR.**


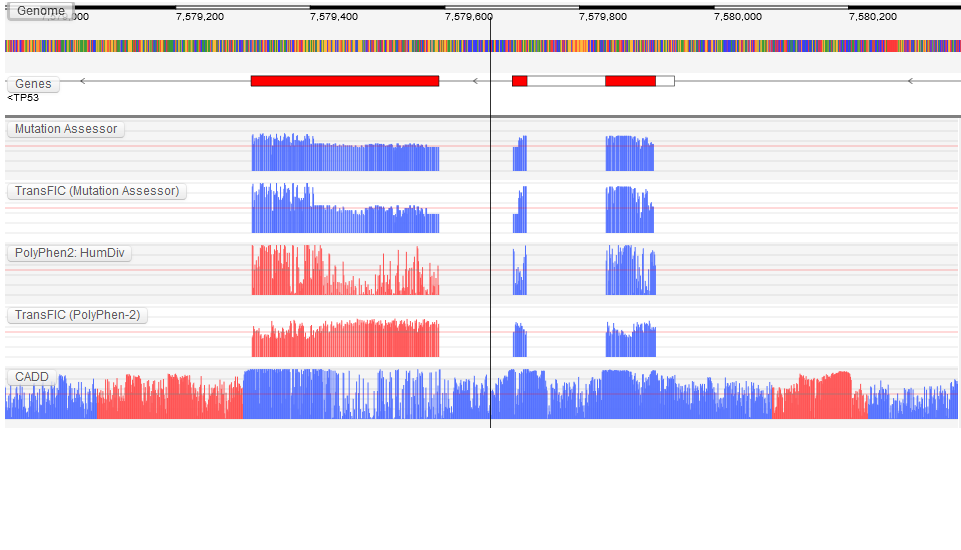


**Figure 3. Differences between generic and cancer-specific prediction algorithms can be observed across TP53. For example, cancer-specific transformations of traditional germline prediction algorithms amplify intolerance across the entire region.**


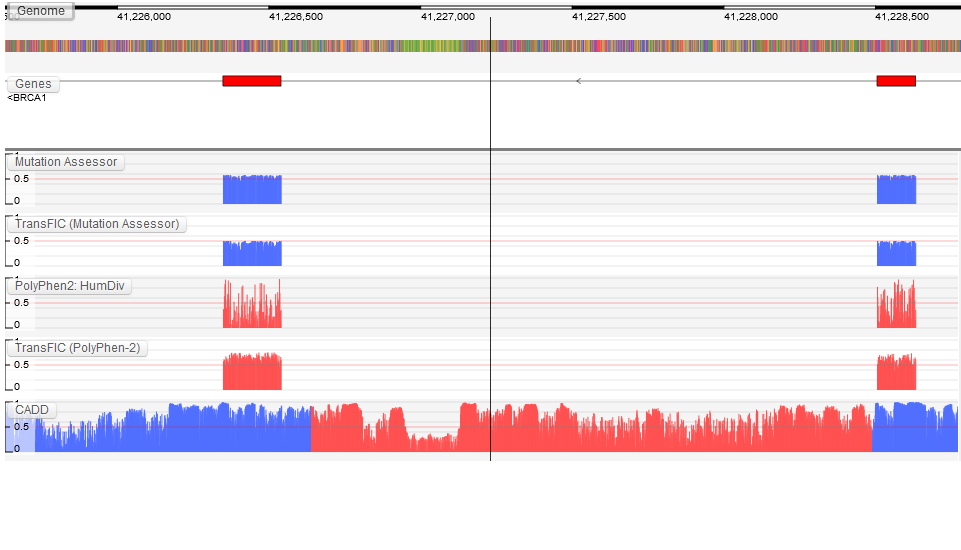


**Figure 4. Cancer-specific transformations of traditional germline prediction algorithms amplify the intolerance of coding regions within BRCA1.**
